# Hyperbaric Oxygenation Prevents Loss of Immature Neurons in the Adult Hippocampal Dentate Gyrus Following Brain Injury

**DOI:** 10.3390/ijms24054261

**Published:** 2023-02-21

**Authors:** Rada Jeremic, Sanja Pekovic, Irena Lavrnja, Ivana Bjelobaba, Marina Djelic, Sanja Dacic, Predrag Brkic

**Affiliations:** 1 Institute of Medical Physiology “Richard Burian”, Faculty of Medicine, University of Belgrade, 11000 Belgrade, Serbia; 2 Department of Neurobiology, Institute for Biological Research “Sinisa Stankovic”, National Institute of the Republic of Serbia, University of Belgrade, 11000 Belgrade, Serbia; 3 Department of General Physiology and Biophysics, Institute of Physiology and Biochemistry, Faculty of Biology, University of Belgrade, 11000 Belgrade, Serbia

**Keywords:** traumatic brain injury, hyperbaric oxygenation, adult neurogenesis, dentate gyrus

## Abstract

A growing body of evidence suggests that hyperbaric oxygenation (HBO) may affect the activity of adult neural stem cells (NSCs). Since the role of NSCs in recovery from brain injury is still unclear, the purpose of this study was to investigate the effects of sensorimotor cortex ablation (SCA) and HBO treatment (HBOT) on the processes of neurogenesis in the adult dentate gyrus (DG), a region of the hippocampus that is the site of adult neurogenesis. Ten-week-old Wistar rats were divided into groups: Control (C, intact animals), Sham control (S, animals that underwent the surgical procedure without opening the skull), SCA (animals in whom the right sensorimotor cortex was removed via suction ablation), and SCA + HBO (operated animals that passed HBOT). HBOT protocol: pressure applied at 2.5 absolute atmospheres for 60 min, once daily for 10 days. Using immunohistochemistry and double immunofluorescence labeling, we show that SCA causes significant loss of neurons in the DG. Newborn neurons in the subgranular zone (SGZ), inner-third, and partially mid-third of the granule cell layer are predominantly affected by SCA. HBOT decreases the SCA-caused loss of immature neurons, prevents reduction of dendritic arborization, and increases proliferation of progenitor cells. Our results suggest a protective effect of HBO by reducing the vulnerability of immature neurons in the adult DG to SCA injury.

## 1. Introduction

After years of debate, it is accepted that adult neurogenesis exists in mammals and new functionally integrated neurons are generated throughout adulthood [1]. Neural stem cells (NSCs) in the adult brain reside in the subventricular zone (SVZ) of the lateral ventricle and the subgranular zone (SGZ) of the dentate gyrus (DG) of the hippocampus [2,3]. Through the lifespan, NSCs in the DG add excitatory granular neurons that can integrate into the neuronal network in the granule cell layer (GCL) [4]. Adult neurogenesis in the DG is thought to significantly increase the neural plasticity of the DG and thereby increasing hippocampal functionality [5].

The characteristics of the neurogenic brain regions that enable the smooth development of adult neurogenesis are unclear. Still, it is known that neurogenesis can be promoted or suppressed by various intrinsic or extrinsic factors [2,6]. Adult neurogenesis increases in response to various brain injuries in both neurogenic regions. A positive correlation has been found between the extent of neurogenesis and recovery after traumatic brain injury (TBI) [5].

It has been extensively reported that TBI is still a global burden on the health care system with its growing age-standardized incidence [7,8]. TBI consists of primary and secondary injuries. Primary injury causes permanent loss of neurons that cannot be repaired. Secondary injury represents neuronal degeneration, which is a consequence of primary injury [9,10,11,12]. Our previously published article suggested that secondary injury should be considered as a chronic non-communicable disease [11]. In light of this, and to alleviate symptoms of TBI, the secondary injury should be a potential target for therapeutic procedures. Currently, studies in animal models of TBI are needed to investigate the exact mechanisms of injury and the recovery process [8].

Patients may have post-traumatic amnesia among a wide range of symptoms after TBI [13]. In addition, because damage to the hippocampus has been shown to result in an inability to form new memories [14], cortex lesions can be expected to indirectly affect the morphology and number of neurons in the DG, a part of the hippocampus.

Given the complexity of TBI, a combination of different therapeutic protocols would likely provide the best results. Hyperbaric oxygen therapy (HBOT) has found its place as a preconditioning treatment or adjunctive therapy in treating TBI [8,15]. HBOT is a therapeutic procedure in which the patient intermittently inhales 100% oxygen at a pressure greater than 1 atmosphere absolute (1 ATA) [16]. Many studies have shown that HBOT has positive and negative effects. Interestingly, under hyperbaric conditions, oxygen can deeply penetrate ischemic regions, which may lead to a reduction in lesions caused by TBI [16]. On the other hand, numerous studies have investigated the oxygen toxicity and oxidative stress that may be caused by HBOT [17]. To avoid the side effects of high oxygen concentrations, the treatment parameters of HBOT, such as pressure and duration, must be controlled [18].

As part of neuroplasticity, neurogenesis and synaptogenesis show that the adult brain can adapt even to TBI [19,20]. To our knowledge, we were the first to report that HBOT applied after TBI increases synaptophysin expression, a marker of synaptogenesis. In addition, improvement in locomotor performance and sensorimotor integration was noted after HBOT [21]. Furthermore, a growing number of data suggests that hyperbaric oxygenation can influence the activity of adult NSCs. In addition, recent studies have shown that HBOT stimulates adult neurogenesis [22,23]. Moreover, HBOT promotes the mobilization of neural stem cells to the lesion site to replace presumably damaged neurons [23]. Although the exact mechanisms of HBOT-induced neurogenesis in adults are still unknown, previous studies suggest that various factors, such as hypoxia-inducible factors, are involved [24].

The aim of this study was to investigate the effects of brain injury induced by sensorimotor cortex ablation (SCA) on DG, and the potential therapeutic impact of HBOT on SCA-induced injury by stimulation of adult mammalian neurogenesis. We found that HBOT could prevent SCA-induced loss of newborn immature neurons and impairment of their morphology. In addition, HBOT increased the number of proliferating cells in hippocampal DG after the SCA injury. To our knowledge, this is the first comprehensive immunohistochemical study to visualize the beneficial effects of HBOT on injury-affected neurogenesis in adult hippocampal DG.

## 2. Results

Notably, there was no statistically significant difference between data obtained for the C and S groups (Figures S2 and S3); therefore, for immunohistochemical and immunofluorescence analysis, all comparisons were made regarding intact controls (data are shown in the Supplemented Material).

### 2.1. SCA Leads to Layer-Specific Neurodegeneration in the Hippocampal DG

**Figure 1:**
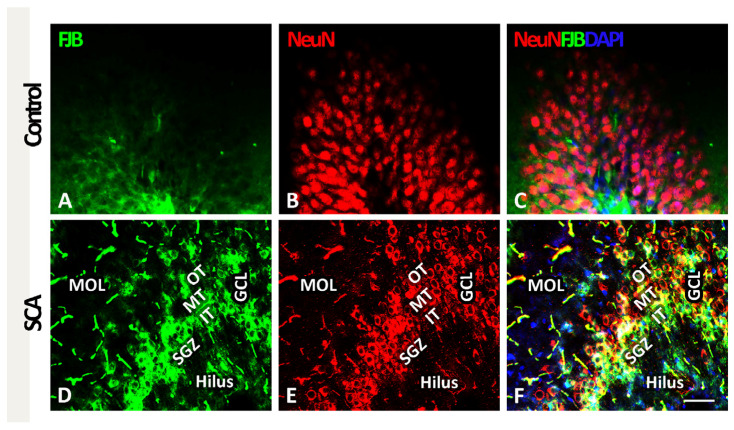
SCA (suction cortical ablation) induces neuronal death predominantly in the inner- and mid-third of the granule cell layer (GCL) and subgranular zone (SGZ) of the dentate gyrus (DG). (**A**–**C**) FJB (green fluorescence) staining in the control sections was weak, and FJB-positive cells were rare. (**D**) After SCA, a substantial increase in FJB-immunoreactivity indicates increased cellular degeneration. (**E**) Reduced NeuN (red) immunoreactivity indicates neuronal loss. (**F**) FJB (green), NeuN (red), and DAPI (blue) fluorescence show strong co-staining, revealing the neuronal identity of degenerating cells. In (**C**,**F**), the sections were counterstained with DAPI (blue) to visualize cell nuclei. Most affected are the inner- and mid-third of the GCL and the SGZ, indicating that neurons are undergoing, degeneration particularly in these compartments. FJB/NeuN-positive neurons were occasionally seen in the molecular layer (MOL) of the DG and the hilus. Outer-third (OT), mid-third (MT), inner-third (IT) of the GCL, and the SGZ of the DG. Scale bar 50 µm.

In order to characterize neuronal death in the hippocampal DG and to visualize the location of the cells undergoing degeneration, we used FJB staining (in green, Figure 1A,D) and NeuN to visualize neurons (in red, Figure 1B,E). Given that the GCL of the DG is further divided into an outer-, mid-, and inner-third of granular neurons and the SGZ, where the NSCs are located [25], we wanted to characterize the impact of SCA on the distribution of the FJB-positive neurons in these specific sub-layers. After analysis, no degenerating neurons were seen in any regions of the DG control sections as assessed using FJB staining (Figure 1A,C). In contrast, SCA caused massive neurodegeneration in the DG, as indicated by an increase in the FJB-immunoreactivity (Figure 1D).

Importantly, in SCA sections, we found the sub-layer specificity in the distribution of the FJB- and NeuN-positive neurons (Figure 1D–F). Co-labeling of FJB and NeuN (see yellow fluorescence in Figure 1F) allowed us to detect degenerating neurons and revealed that the vast majority of these NeuN/FJB-positive neurons were located in the inner- (IT) and mid-third (MT) of the GCL and partially in the SGZ. On the other hand, FJB/NeuN-positive neurons were rarely detected in the outer-third (OT) of the GCL of the DG. Furthermore, only a few FJB/NeuN-positive neurons were found in the molecular layer (MOL) of the DG and the hilus (Figure 1F). Based on these results, we concluded that SCA has layer-specific effects on the DG, causing cell death of neurons predominantly in the inner GCL and the SGZ.

### 2.2. HBOT Prevents/Ameliorates SCA-Provoked Loss of Neurons in the GCL of the Hippocampal DG

**Figure 2:**
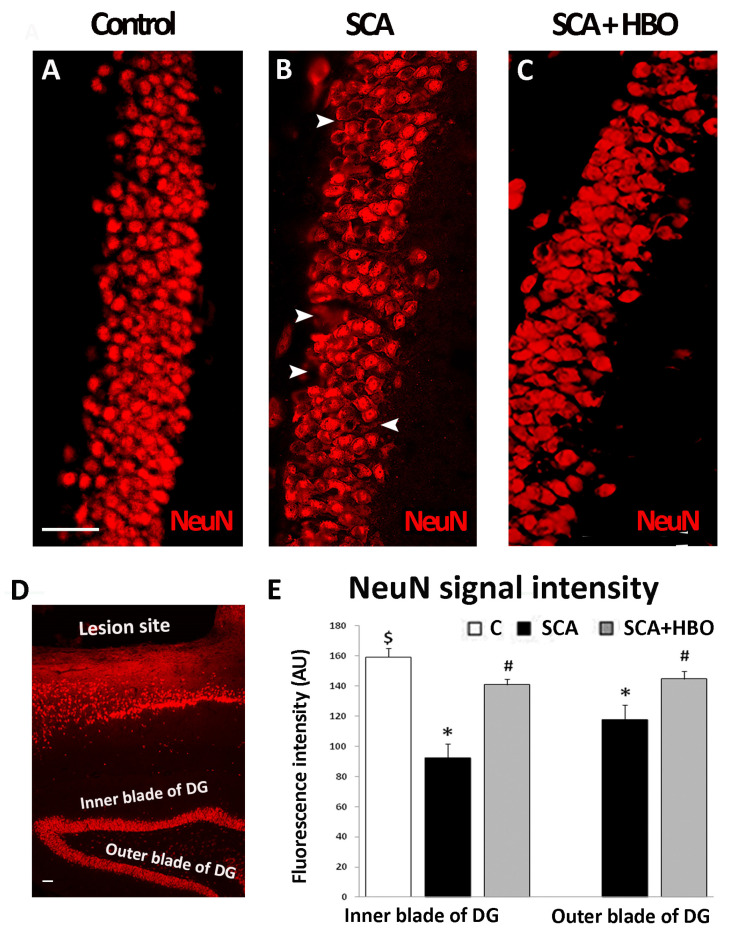
Effect of SCA (suction cortical ablation) and HBOT (hyperbaric oxygen therapy) on NeuN immunoreactivity in the GCL (granule cell layer) of the hippocampal DG (dentate gyrus). Significant loss of granular neurons stained with NeuN (red fluorescence) is seen in the DG after SCA (**B**, arrowheads) in comparison to the control sections (**A**). In contrast, (**C**) HBOT ameliorated neuronal loss. (**D**) NeuN staining in the hippocampus underlying the lesion site. (**E**) NeuN fluorescence signal intensity (in arbitrary units, AU), quantified separately in the inner and outer blades of the DG in controls (**C**, white bar), SCA (black bars), and HBO (hyperbaric oxygenation)-treated animals (SCA + HBO, gray bars). After the SCA, the brain sections show a statistically significant decrease in NeuN signal intensity in the inner blade and, to a less extent, in the outer blade of the DG compared to the controls. Following HBOT, the signal intensity of NeuN was comparable to the control level. C—Control, SCA—Suction cortical ablation, SCA + HBO—SCA animals treated with hyperbaric oxygen. Bars represent mean ± SD. The level of significance was analyzed using One-way ANOVA with Tukey’s multiple comparisons post hoc test (* *p* < 0.001 SCA vs. C, $ *p* < 0.01 SCA + HBO vs. C, # *p* < 0.001, SCA + HBO vs. SCA). Scale bar 50 µm (**A**–**D**).

To determine whether HBOT could prevent or at least ameliorate SCA-induced cellular loss in the hippocampal DG granule neurons, we performed immunofluorescence staining with neuronal marker NeuN. Cortical injury caused a significant loss of granular neurons stained with NeuN (red fluorescence) in the DG (Figure 2B, arrowheads) in comparison to the control sections (Figure 2A). After SCA, 10 successive HBO treatments prevented/ameliorated neuronal death after SCA (Figure 2C).

Since the loss of NeuN immunoreactivity may predict neuronal degeneration in the rodent hippocampus after various brain injuries [26], we quantified separately NeuN labeling in the suprapyramidal (inner) blade and infrapyramidal (outer) blade of the hippocampal DG [27] in coronal sections of the control, SCA, and HBO-treated animals (Figure 2D,E).

In the control sections, there was no significant difference (*p* = 0.114) in the NeuN fluorescence intensity (in arbitrary units, AU) between the inner (159.21 ± 6.18) and outer (153.79 ± 4.56) blades of the DG (Figure S2). Hence, all comparisons were made against the NeuN fluorescence intensity of the inner blade.

SCA caused a significant reduction (41.85%, *p* < 0.001) of NeuN fluorescence intensity (92.59 ± 9.25, black bar) in the inner blade of the DG compared to controls (159.21 ± 6.18, white bar). However, after 10 HBOT, this SCA-induced neurodegeneration was less pronounced (11.49%, *p* < 0.01) (140.93 ± 3.92, gray bar).

In the outer blade of the DG of animals exposed to SCA, the reduction of NeuN fluorescence intensity was lesser but still significant (26.02%, *p* < 0.001) (117.78 ± 9.52, black bar) vs. the signal intensity of the control sections (159.21 ± 6.18, white bar). On the other hand, in the HBOT sections, the effect of SCA was attenuated and no statistically significant (9.04%, *p* = 0.227) difference was observed in the NeuN fluorescence intensity (144.82 ± 5.02) (Figure 2E, gray bar) vs. control. Together, these results suggest that SCA injury induces massive neuronal degeneration in the inner and outer blades of the DG, judging by the significant loss of NeuN immunoreactivity. In contrast, HBOT almost completely prevents/ameliorates this.

### 2.3. Cell Type of Neurons Undergoing Neurodegeneration in the Hippocampal DG Following SCA Injury and HBOT

Given that a large amount of FJB-positive neurons were detected in the SGZ layer, where the NSCs reside, in this section, we further performed immunofluorescence staining with doublecortin (DCX, green), a marker for early newborn immature neurons [28] and beta-III tubulin (TUJ1, red), which is expressed during hippocampal neurogenesis after DCX and marks newly generated postmitotic neurons [29,30].

#### 2.3.1. HBOT Prevents Loss of DCX-Positive Newborn Immature Neurons in the GCL of the Hippocampal DG Following SCA Injury

**Figure 3:**
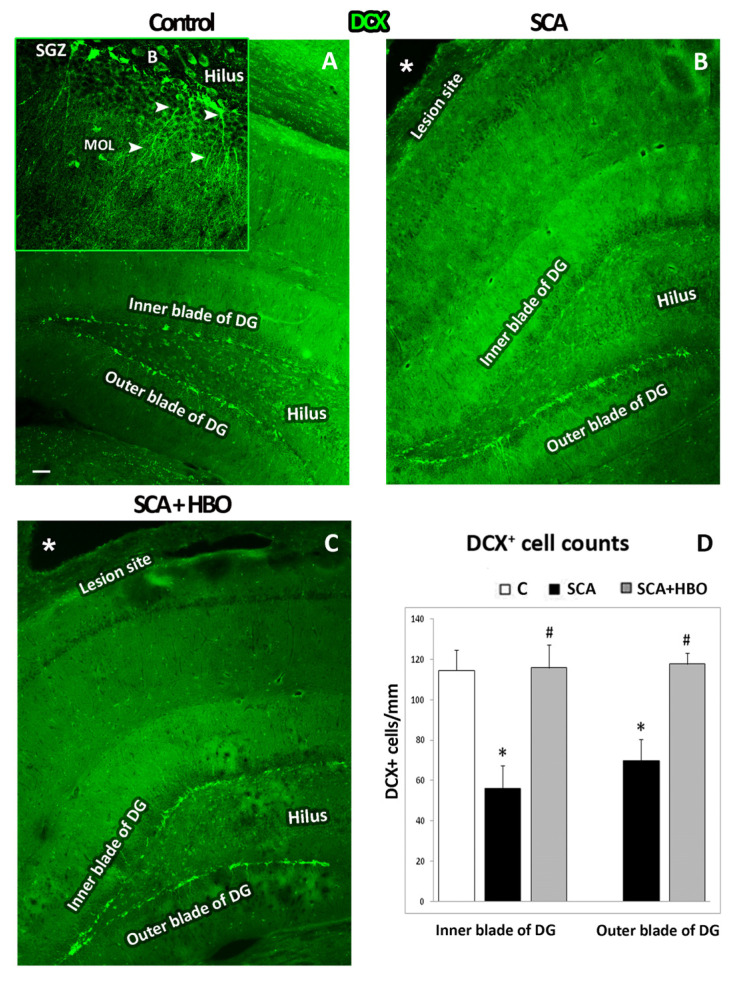
HBOT prevented the loss of doublecortin (DCX)-immunopositive newborn immature neurons after SCA (suction cortical ablation) in the subgranular zone (SGZ) of the hippocampal dentate gyrus (DG). A total of 25 µm thick frozen brain sections of C—Control, SCA—Suction cortical ablation, and SCA + HBO—SCA animals treated with hyperbaric oxygen were stained with DCX (green). (**A**) In the control sections, intensive DCX-immunopositivity was seen in the SGZ of the inner and outer blades of the DG. At higher magnification (inset), intensively DCX-labeled newborn neurons with branched dendrites (arrowheads) extended from the SGZ until the molecular layer (MOL). In addition, DCX immunoreactivity is detected in progenitors and basket cells (**B**) in the hilus. (**B**) SCA reduced DCX-immunofluorescence and was particularly pronounced in the inner blade of the DG beneath the lesion site (asterisk). (**C**) After 10 successive HBOT (hyperbaric oxygen therapy), the level of DCX-immunopositivity was as observed in the control. (**D**) DCX-positive cells were counted separately in the SGZ of the inner and outer blades of the hippocampal DG. Bars represent mean ± SD. Control (C, white bar), SCA (black bars), and HBO (hyperbaric oxygenation)-treated animals (SCA + HBO, gray bars). The significance level was analyzed using One-way ANOVA with Tukey’s multiple comparisons post hoc test (* *p* < 0.001 SCA vs. C, # *p* < 0.001 SCA + HBO vs. C). Scale bar: (**A**–**C**)—100 µm.

First, we performed immunofluorescence staining with doublecortin (DCX, green) to identify the cell layer in which these early newborn immature neurons are localized, as well as to count the number of DCX-positive neurons in the SGZ layer of control, SCA, and HBOT sections separately in the inner and outer blades. As it is apparent from Figure 3, most of the DCX immunoreactivity was found in the SGZ layer. In the control sections (Figure 3A), there was no statistically significant (*p* = 0.191) difference between the number of DCX-positive cells in the inner (114 ± 10.31) and outer (120 ± 5.14) blades of the DG (Figure S3). Therefore, all comparisons were made against the number of DCX-positive cells in the inner blade (Figure 3D, white bar). DCX immunoreactivity is mainly seen in the SGZ in DCX-positive granular neurons with dendrites that elongate from the SGZ until MOL and in the cells located on the hilar border of the granular layer, probably basket cells (Figure 3A, inset).

**Figure 4:**
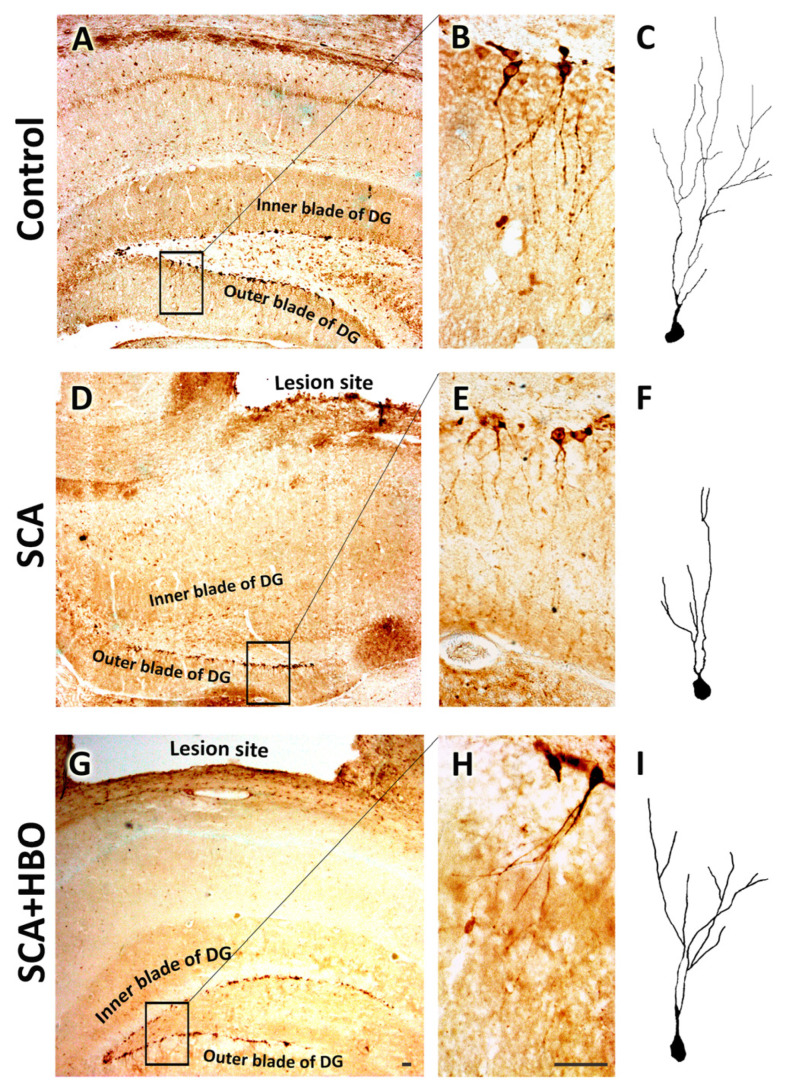
Besides prevention of SCA (suction cortical ablation)—which caused the neuronal loss, HBOT (hyperbaric oxygen therapy) impends dendrite degeneration of immature neurons in the SGZ (subgranular zone) as well. (**A**,**B**) In the control sections, DCX-stained immature neurons are in the SGZ, with dendrites branching in the inner- and mid-third of the granule cell layer (GCL) until the molecular layer. (**C**) The reconstructed neuron in the control group. (**D**,**E**) SCA causes the loss of neurons in the inner blade. (**E**) Higher magnification of the outer blade reveals that neurons in the SGZ show significant morphological changes manifested by dendritic shrinkage and reduction of arborization. (**F**) Reconstructed neuron after SCA. (**G**,**H**) HBOT protects neurons in the SGZ and prevents dendritic degeneration. (**I**) Reconstructed neuron following HBOT. Rectangles indicate where the high-magnification images are taken. Scale bars: (**A**,**D**,**G**)—50 µm, and (**B**,**C**,**E**,**F**,**H**,**I**)—10 µm.

SCA caused a significant (*p* < 0.001) loss in the number of DCX-positive immature neurons, particularly in the inner blade (50.90%) (56 ± 11.03, black bar) compared to the controls (114 ± 10.31, white bar) (Figure 4B,D), and to a less extent, but still noteworthy (38.90%, *p* < 0.001) in the outer blade (70 ± 10.58, black bar).

Ten successive HBOTs prevented the loss of DCX-positive newborn immature neurons in the SGZ (Figure 3C). In addition, the number of DCX-positive cells in both the inner (116 ± 11.48) and outer (118 ± 5.63) blades of the hippocampal DG (Figure 3D, gray bars) was similar to those counted in control animals. These data indicate that newborn neurons are particularly vulnerable to SCA, while HBOT was able to overcome these effects of SCA and protect these newborn neurons from death.

#### 2.3.2. HBOT Prevents SCA-Caused Neuronal Loss and Dendrite Degeneration of Newborn Immature Neurons in the SGZ of the Hippocampal DG

Light microscopic analysis of DCX immunostaining confirmed a significant loss of DCX-stained cells in the inner blade of the DG beneath the site of the lesion (Figure 4D) compared to the control sections (Figure 4A). Moreover, SCA caused morphological alterations of immature neurons in the SGZ layer of the inner and outer blade (Figure 4E,F). Higher-resolution images of the DG revealed that dendrites of spared neurons in the SGZ were damaged and underwent significant morphological changes. Namely, SCA induced an extreme reduction of dendritic complexity of SGZ neurons, which was manifested by the shortening of dendrite length and reduction of dendritic arborization (Figure 4E,F), as compared with controls (Figure 4B,C).

HBOT prevents and ameliorates these SCA-induced morphological alterations of neurons (Figure 4G–I), and these immature neurons resemble those in the control sections.

In order to show the effect of SCA and HBOT on dendrite arborization, we compared the dendrite total length, average segment length, and the number of branching points of the neurons in the outer blade between the groups (Table 1). There were significant decreases in the dendrite total length (by 43.09%, *p* < 0.001) and the number of branching points (by 60.77%, *p* < 0.001) in the SCA group compared to the control and an increase in the average segment length (by 33.18%, *p* < 0.01). According to this, SCA caused a dramatic reduction of dendritic arborization of the immature neurons in SGZ.

In the SCA + HBO group, the total length of the dendrites was also reduced, but to a lesser extent (by 18.99%, *p* < 0.01), as well as the number of branching points (by 26.3%, *p* < 0.01) compared to the control group. However, values of average segmental length were similar in these two groups (*p* > 0.05).

Altogether, these results suggest that SCA reduces the number of newborn neurons in the DG and causes significant impairment in the development and dendritic arborization. In contrast, HBOT attenuates these changes and protects the morphology of these newborn neurons.

#### 2.3.3. HBOT Prevents Loss of DCX/TUJ1-Positive Newborn Immature Neurons in the GCL of the Hippocampal DG Following SCA Injury

**Figure 5:**
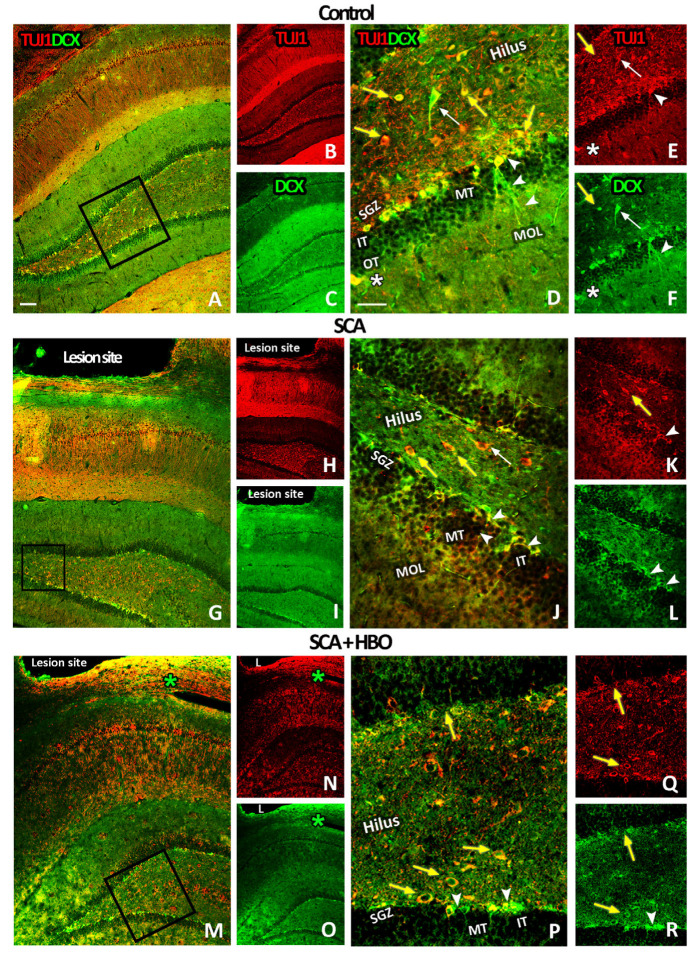
DCX (doublecortin) and TUJ1 (beta-III tubulin) immunoreactivity in the hippocampal DG (dentate gyrus) of control, SCA (suction cortical ablation), and HBO (hyperbaric oxygenation)-treated SCA animals. DCX (green) and TUJ1 (red) staining were used to visualize newborn neurons in the brain sections from controls (**A**–**F**), animals undergoing SCA (**G**–**L**), and HBOT (hyperbaric oxygen therapy) (**M**–**R**). (**D**–**F**) Higher magnification images of control sections revealed DCX/TUJ1-positive cells in the SGZ (subgranular zone), IT (inner-third) of the GCL (granule cell layer), with dendrites extending toward the MT (mid-) and OT (outer-third) of the GCL (arrowheads). A few DCX/TUJ1-positive cells were located in the OT of the GCL and molecular cell layer (MOL) (white asterisks). In the hilus, we detected some intensely stained DCX/TUJ1-positive cells with large cell bodies (white arrows) and others with round/oval morphology (yellow arrows) that were mostly TUJ1-positive (**J**–**L**). Higher magnification indicates that SCA reduced the number of DCX/TUJ1-positive immature neurons in the SGZ compared to control sections (**D**–**F**). (**M**–**O**) After HBOT, concentrated DCX/TUJ1 immunoreactivity is seen around the lesion site (green asterisks), (**P**–**R**) in the SGZ (arrowheads) and in the hilus (yellow arrows) as well. Rectangles indicate where the high-magnification images are taken from. C—control; SCA—sensorimotor cortex ablation. SCA + HBO—SCA animals treated with hyperbaric oxygen. Scale bars: (**A**,**G**,**M**)—100 µm; (**D**,**J**,**P**)—50 µm.

To confirm the results mentioned above, we next performed double immunofluorescence staining with the most accepted markers for early neurons: doublecortin (DCX, green), a marker of early newborn immature neurons in adult DG, and beta-III tubulin (TUJ1, red), which marks newly generated postmitotic neurons (Figure 5). As expected, double staining revealed that in the DG of control sections, DCX/TUJ1-positive immature neurons were mainly located in the SGZ layer of the inner and outer blades (Figure 5A–C). At the higher magnification, DCX/TUJ1-positive cells were also visible in the IT of GCL, with their dendrites extending toward the MT and OT of GCL (Figure 5D–F, arrowheads). Notably, neuronal cell bodies were intensively stained both with DCX and TUJ1 (Figure 5D–F), while dendrites were stained only with DCX (Figure 5D,F, arrowheads). Only a few DCX/TUJ1-positive cells were in the OT of GCL and molecular cell layer (MOL) (Figure 5D–F, white asterisks). In the hilus, a paucity of intensely stained DCX/TUJ1-positive cells with large cell bodies were detected (Figure 5D–F, white arrows), while others with round/oval morphology were mostly TUJ1-positive (Figure 5D–F, yellow arrows).

SCA reduced the DCX/TUJ1-immunoreactivity in the SGZ (Figure 5G–I), principally in the inner blade of the DG below the lesion site. The remaining DCX/TUJ1-positive neurons had altered morphology with shortened and less-branched dendrites (Figure 5J–L, arrowheads). In the hilus, TUJ1-positive neurons with round/oval morphology were still predominant (Figure 5J,K, yellow arrows).

Ten repetitive HBOTs prevented the loss of DCX/TUJ1-positive immature neurons mainly located in the SGZ layer of the inner and outer blade (Figure 5M–O). Interestingly, at higher magnification, we detected that besides neurons with round/oval morphology, which were only TUJ1-positive, some of them were also DCX/TUJ1-positive (Figure 5P–R, yellow arrows). Furthermore, these DCX/TUJ1-positive neurons were primarily located at the hilar border of the GCL. Taken together, these data indicate that newborn neurons were especially vulnerable to SCA, particularly in the inner blade facing the lesion site. Moreover, it is noteworthy to mention that after HBOT, a robust increase of DCX/TUJ1-immunoreactivity, which was widely distributed around the lesion site, was seen (Figure 5M–O, green asterisks).

### 2.4. Proliferation of Ki67-Positive Newborn Immature Neurons Co-Labeled with DCX in the SGZ of the Hippocampal DG Following SCA Injury and HBOT

**Figure 6:**
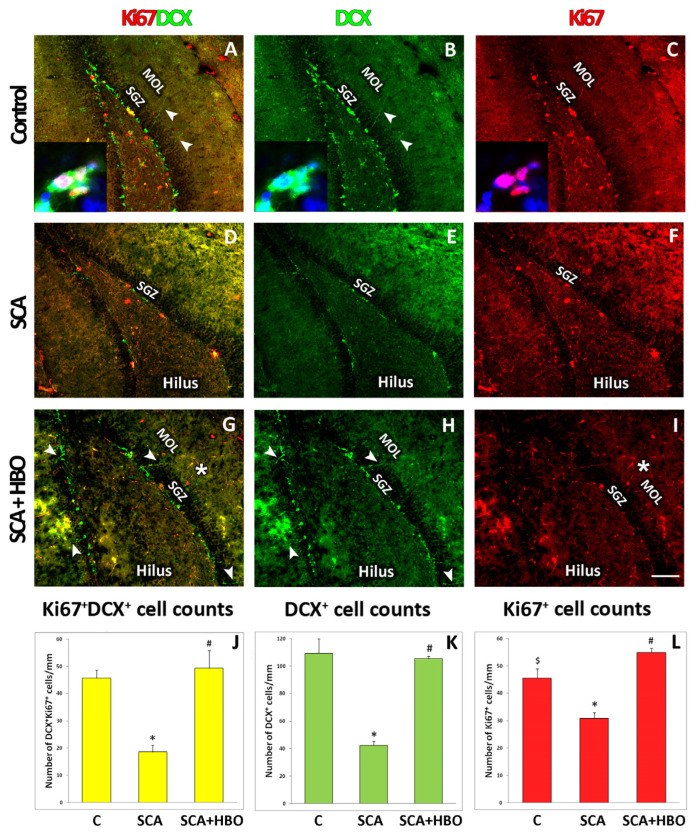
Effect of SCA (suction cortical ablation) and HBOT (hyperbaric oxygen therapy) on cell proliferation in the hippocampal DG (dentate gyrus). The proliferation marker Ki67 was used to quantify the number of dividing cells (red fluorescence), while DCX (a marker of immature neurons, green fluorescence) was used to identify cells of neuronal lineage. The higher magnification images of control sections were counterstained with DAPI (blue) to visualize cell nuclei. (**A**–**C**, inset) The Ki67 signal was restricted to the nuclei of cells (red fluorescence, **A**,**C** insert). In contrast, (**A**,**B**, inset), the DCX signal was mainly located in the cell cytosol (green fluorescence) and the cellular processes arising from the SGZ (subgranular zone) until the MOL (molecular cell layer) (green fluorescence, (**A**,**B**, arrowheads). SCA reduced Ki67+/DCX+ (**D**, yellow fluorescence), DCX+ (**E**, green fluorescence), and Ki67+ (**F**, red fluorescence) immunoreactivity in SGZ. In contrast, HBOT increased the number of DCX+ neurons with processes extending until the MOL (**G**,**H** arrowheads) and increased Ki67 cells in SGZ, hilus, and MOL (**I**, red fluorescence, asterisk). C—Control, SCA—Suction cortical ablation, SCA + HBO—SCA animals treated with hyperbaric oxygen. (**J**–**L**) Cells that were Ki67+/DCX+ (yellow fluorescence), DCX+ (green fluorescence), and Ki67+ (red fluorescence) were counted along the entire SGZ of the DG. While the number of all counted cells was drastically reduced after SCA, following HBOT, the number of proliferating immature neurons was at the same level as observed in the controls. In contrast, the overall proliferation of all Ki67-positive cells increased. Bars represent mean ± SD. The level of significance was analyzed using One-way ANOVA with Tukey’s multiple comparisons post hoc test (* *p* < 0.001 SCA vs. C, $ *p* < 0.001 SCA + HBO vs. C, # *p* < 0.001 SCA + HBO vs. SCA). Scale bar 50 µm (**A**–**I**).

To evaluate the proliferative cells of the neuronal lineage in the SGZ of the hippocampal DG after the SCA and HBOT, we performed double immunofluorescent staining with Ki67 (a marker of cell division, red fluorescence), and DCX (a marker of immature neurons, green fluorescence). We counted the number of cells with ongoing proliferation along the entire length of the SGZ of the DG in control, SCA, and SCA + HBO brain sections. Counted cells were either Ki67+/DCX+ (yellow fluorescence, Figure 6A,D,G,J), DCX+ (green fluorescence, Figure 6B,E,H,K), or Ki67+ (red fluorescence, Figure 6C,F,I,L). The results revealed that in all the investigated groups, the majority of cells were DCX-positive. Ki67-positive cells were located predominantly in the SGZ and hilus, irrespective of the investigated group. As expected, the Ki67 signal was restricted to the nuclei of cells (red fluorescence, Figure 6A,C, inset). In contrast, the DCX signal was found mainly in the cell cytosol (green fluorescence, Figure 6, inset to A,B) and the cellular processes arising from the SGZ until the MOL (green fluorescence, Figure 6A,B,G,H, arrowheads). It is important to note that HBOT increased the number of neurons with processes protruding until the molecular cell layer (Figure 6G,H) in contrast to SCA sections (Figure 6D,E), where this was only occasionally seen. Moreover, HBOT increased Ki67-stained cells in the SGZ, hilus, and MOL (Figure 6I, asterisk). When quantified, we demonstrated that SCA radically reduced the number of all counted cells. Compared to the control, the number of Ki67+ cells co-expressing DCX+ decreased by 58.7% (yellow fluorescence, Figure 6J), DCX+ cells by 61.47% (green fluorescence, Figure 6K), and Ki67+ cells by 31.11% (red fluorescence, Figure 6L). In contrast to SCA, after HBOT, the number of Ki67-expressing DCX-positive progenitors was slightly (6.52%) increased compared to controls (yellow fluorescence, Figure 6J), while the number of Ki67+ cells was increased by 22.22% (red fluorescence, Figure 6L). The number of DCX+ cells in the SCA + HBO group was slightly decreased (3.67%) vs. the control group. Next, we determined the fraction of dividing Ki67-expressing progenitors in all the investigated groups. In all the investigated groups of animals, control, SCA, and SCA + HBO, almost the same number of DCX-positive cells co-expressed Ki67 (42.2%, 45.2%, and 46.7%, respectively), being the largest in the HBOT group, where around half of all DCX-positive cells were proliferating immature neurons (Ki67+/DCX+). These results suggest that SCA reduced the number of proliferating and total DCX-expressing progenitors and all Ki67-positive cells. In contrast, HBOT increased the number of proliferating cells after the SCA injury.

## 3. Discussion

Considering that adult hippocampal neurogenesis is restricted to only one part of the hippocampal formation, the dentate gyrus—DG [31], in this article, we investigate how hyperbaric oxygenation overcomes the impairments of neurogenesis in the adult DG caused by brain injury. The results show that our model of experimental cortical trauma, ablation of the sensorimotor cortex, causes a loss of DG neurons, mostly in the inner granular neuron layer of the inner blade underlying the lesion site. Analysis with cell-specific markers shows that mostly immature neurons of the subgranular layer of DG degenerate. We also demonstrate that injury leads to a reduction in dendrites branching of spared neurons. The most striking finding of this study is that HBOT prevents SCA-induced neuronal death in both the inner and outer blades of the granular layer of the DG. In addition, HBOT prevents the degeneration of dendrites and significantly reduces the loss of newborn immature neurons. Finally, using the endogenously expressed marker Ki67 to label and detect dividing cells and the marker of neuronal progenitors doublecortin (DCX), we demonstrate that SCA radically reduces the number of proliferating cells in SGZ, particularly those of neuronal lineage. Conversely, HBOT increases overall cell proliferation after SCA.

The experimental results on the effects of TBI on hippocampal neurogenesis are complex and seemingly contradictory [32,33,34,35,36]. An important factor contributing to these discrepancies is that different TBI models have other effects on neurogenesis in the adult hippocampus. To better understand how brain trauma can affect the generation of new neurons, which is a prerequisite for using this process to enhance brain repair, it is essential to examine the effects of different TBI models on hippocampal neurogenesis. For methodological reasons, many studies of the effects of TBI on the hippocampus focus on degenerating cells, changes in neurogenesis, and other morphological alterations that can be visualized by using immunostaining or other standard morphological techniques. The specific TBI lesion model, the suction-ablation of the sensorimotor cortex (SCA), that we use here has been thoroughly characterized previously [21,37,38]. SCA is a well-characterized model of focal traumatic brain injury, which permits highly reproducible lesions in the hindlimb sensorimotor cortex, uniform in size and depth [39]. The advantages of this type of cortex injury are well described in Goldstein’s study [37]. Since the lesion is the result of actual removal of brain tissue, pathological events such as inflammatory responses and reactive gliosis are limited [39,40], while secondary processes associated with other types of injuries such as ischemia [41,42], concussive trauma [43], and electrolytic lesions [39] are minimized [37]. This type of injury mimics a clinical condition of immediate brain tissue removal, such as during surgical removal of brain tumors [44].

In our recently published paper, we applied the gray-level co-occurrence matrix algorithm for textural analysis of granular cell bodies to show that SCA resulted in subtle morphological changes in hippocampal DG neurons that could not be detected via classical immunohistochemical analysis [20]. In the present study, we report that SCA causes damage of hippocampal DG neurons, as shown by the reduction of NeuN immunoreactivity, which was used to predict neuronal degeneration in the rodent hippocampus after various brain injuries [26]. The extensive loss of granule cell neurons in this region indicates that the loss of neurons is the most prominent in the part of the granule cell layer below the lesion site. These changes in the GCL probably contribute to the injury-induced impairments of locomotor coordination observed in our previous publications [21,38,45]. Having found that SCA leads to a substantial loss of hippocampal cells, as shown by thinning of the neuronal layers, we want to specify which part of the GCL is most affected. In this way, our results reveal that SCA leads to specific damage of the hippocampus and predominantly affects neurons in the inner-third layer of the inner blade of the GCL. Our findings are consistent with the results of other studies on different animal models of TBI, which also reported that hippocampal neurons are particularly vulnerable to brain injury [32,35,46,47,48]. On the other hand, Becerra et al. [49] have recently shown that controlled cortical impact (CCI) injury causes the loss of neurons in the CA3 region and relative preserves neurons in the GCL, but they did not quantify NeuN+ cells in this region as we did. Indeed, we quantified NeuN fluorescence intensity in the suprapyramidal (inner) and infrapyramidal (outer) blades of the hippocampal DG of control, SCA, and HBO animals and found massive neuronal loss in both the inner (42%) and outer (26%) blades of the DG after SCA injury. In contrast, after HBOT, this effect of SCA is almost completely attenuated. Our results are consistent with those of Baratz and colleagues, who found that HBOT prevents neuronal loss in the blades of the DG [50].

The distribution and extent of cell death in the hippocampus have been shown to vary depending on the injury model [46,51,52], severity of injury [53,54], and age [55]. Moreover, many studies have shown that neurogenesis increases in a time-dependent manner after brain injury depending on the severity of injury [56,57]. Interestingly, our model eliciting a relatively extensive injury shows a similar specific impairment of adult hippocampal neurogenesis as more moderate CCI injury models [32,58]. By combining FJB-staining and double immunofluorescence staining with specific cell-type markers, we demonstrate that predominantly newborn neurons of the SGZ of the hippocampal DG are affected by SCA. To confirm that these progenitor cells are of neuronal lineage, we use DCX, a marker for putative newborn immature neurons [28]. The quantification of DCX-expressing cells shows that the number of DCX-positive immature neurons is significantly reduced (by more than 50%) in the inner blade compared with the control animals and to a lesser extent, but still remarkably (by about 40%) in the outer blade of the DG after the SCA. Our observations are in line with those of other authors who also reported that DCX-expressing neural progenitors are vulnerable to brain injury and undergo cell death in the ipsilateral DG [32,34,36,58]. Light microscope examination of stained sections revealed that spared DCX-positive immature neurons, despite their survival, exhibit a substantial injury-induced alteration of their morphology, manifested by dendritic shrinkage and a considerable reduction in dendritic arborization. Similarly, Villasana et al. also found abnormal dendritic branching after TBI [59]. This significant dendrite damage, accompanied by a reduction in dendritic spines, may represent a potential anatomical substrate that explains, at least in part, the development of posttraumatic memory deficits [35,48]. In contrast, we have shown that 10 consecutive HBOT prevents the loss of these DCX-expressing neural progenitors, ameliorates the observed SCA-caused changes in the SGZ of DG, and protects their morphology. Taken together, our data suggest that HBOT can overcome the harmful effects of SCA and protect newborn neurons from death and morphological deterioration.

To identify the cell type of neurons undergoing degeneration in the SGZ layer of hippocampal DG, we performed double immunofluorescence staining using DCX as a marker for early newborn immature neurons and beta-III tubulin (TUJ1), which is expressed during hippocampal neurogenesis after DCX and labels newly formed postmitotic neurons [29,30]. After the SCA injury, the reduced DCX/TUJ1-immunoreactivity in the SGZ of the DG inner blade facing the lesion site confirms our observations mentioned above that SCA triggers selective death of immature neurons. We also demonstrate that the development of newborn DCX/TUJ1-positive neurons occurs not only in the SGZ layer of the DG but also in the inner- and outer-thirds of the granule cell layer. Interestingly, in the hilus, we found that in addition to the neurons with round/oval morphology, which were only TUJ1-positive, some of them were also DCX/TUJ1-positive. These DCX/TUJ1-positive neurons are primarily located at the hilar border of the granule cell layer and deep in the hilus and are present in all the groups regardless of the treatment protocol. It is suggested that cells with round/oval shape are neuroblasts that are generated in the hilus and migrate to the SGZ and inner part of the GCL to increase the population of neuronal progenitors [60]. They proposed that a substantial population of these hilar progenitors should differentiate into proliferative neuroblasts and immature neurons within the hilus, probably via transitional intermediate cells expressing both astrocytic and neuronal markers. Ten repetitive HBOTs prevent the loss of DCX/TUJ1-positive immature neurons located in the SGZ layer of the inner and outer blades and increase the appearance of progenitors in the hilus. In addition, it is important to note that, after HBOT, DCX/TUJ1-immunoreactivity is abundantly distributed around the lesion site, suggesting that HBOT also increases the number of neuronal progenitors in the peri-lesioned region. However, it is unclear whether these newly generated neurons proliferate locally at the injury site and/or migrate from neurogenic regions along migratory pathways, extending from the SVZ or SGZ to the lesion site, as suggested by some authors [34,61,62].

Finally, we quantify the number of proliferating cells [4] after SCA and HBOT in the subgranular zone of the DG. Immunostaining analysis reveals radically reduced DCX/Ki67-positivity after SCA, particularly in the inner blade of the DG below the lesion site. These observations confirm the results of cell population quantification, showing that after SCA, the fractions of actively dividing neuronal precursors (Ki67/DCX double-positive) and total DCX-expressing progenitors are significantly reduced (by 60%) in the ipsilateral DG. Interestingly, the number of Ki67-labelled cells is reduced to a lesser extent, indicating that some cell populations are unaffected by SCA. These cells are mainly located in the hilus, and most of them do not co-express DCX, suggesting they probably belong to the glial lineage. Our assumption is consistent with the results of Colicos et al. [47], who found that brain injury does not affect the number of astrocytes and oligodendrocytes. Moreover, Liu et al. [63] suggested that the glial fibrillary acidic protein (GFAP)-positive progenitors in the SGZ of the DG give rise to neuronal progenitors that develop into granule neurons. In contrast to SCA, HBOT administration increases overall cell proliferation in the DG, with the proportion of DCX+/Ki67+ proliferating immature neurons accounting for approximately 50% of all DCX-expressing progenitors. Our results are consistent with the observations of Wei et al. [64], who reported that hyperbaric oxygenation promotes neural stem cell proliferation and protects learning and memory in neonatal hypoxic-ischemic brain damage.

Since our knowledge about the exact mechanisms by which HBOT exerts its beneficial effects still needs to be improved, in our recently published review [16], we summarized up-to-date results of potential cellular and molecular mechanisms underlying the beneficial effects of HBOT. We hypothesize that many of these cellular and molecular mechanisms and signaling pathways work in parallel or together, contributing to the establishment of a stimulating local environment that enhances neurogenesis, thereby allowing tissue repair and the recovery of impaired brain functions.

In conclusion, this study shows that SCA not only causes neuronal loss, but also induces remarkable dendritic degeneration of spared neurons and a reduction in proliferation of progenitor cells. In contrast, treatment with HBO prevents the loss and morphological deterioration of immature neurons, promotes the overall proliferation of progenitors, and thus has the potential to improve neurogenesis in the adult hippocampal DG affected by TBI. According to the literature and our results, adequate rehabilitation of the TBI consequences requires a combination of different therapeutic procedures, among which hyperbaric oxygenation therapy seems promising. However, underlying mechanisms remain to be determined.

## 4. Materials and Methods

### 4.1. Animals

The experiment was performed on male Wister albino rats, which were 10 weeks old. Animals were housed in four per cage under standard environmental conditions (23 ± 2 °C, 50–60% relative humidity, 12:12 h light-dark cycle, and food and water ad libitum). Animals were randomly divided into groups: Control group (C; n = 6)—age-matched intact animals; Sham control (S; n = 6)—the rats that underwent the surgical procedure without opening the skull; SCA group (SCA; n = 7)—suction ablation of the right sensorimotor cortex; HBO group (SCA + HBO; n = 6)—the rats that were subjected to the HBO protocol after SCA. There was no significant difference within and between the groups considering the animal body weight (250 ± 30 g). Experimental procedures were approved by the Ethical Committee of the University of Belgrade (No. 61206-2915/2-20). They were carried out in strict accordance with Directive 2010/63/EU on the protection of animals used for scientific purposes. Furthermore, all potential problems were considered to keep animal suffering to a minimum.

### 4.2. Surgical Procedure

Our previously published work describes the surgical procedure in detail [21]. Before the surgery, the rats were anaesthetized with an intraperitoneal injection of Zoletil^®^50 (Virbac, Carros, France) at 50 mg/kg body weight. After providing the anesthesia, rats were shaved and placed into the stereotaxic frame. The scalp was cut with a scalpel along the midline to expose the bregma. The craniotomy coordinates were: 2 mm anterior to the bregma, 4 mm posterior to the bregma, and 4 mm lateral from the midline [64]. The suction ablation of the right sensorimotor cortex was carefully carried out through a polypropylene tip to the depth of white matter with the purpose of keeping the white matter layer intact, thus separating the lesion cavity from the underlying hippocampus. Dura and the bone flap were returned to the place, and the skin was sutured. After the surgery, the rats from the SCA + HBO group were left to recover for up to 5 h before the hyperbaric oxygen treatment.

### 4.3. Hyperbaric Oxygen Treatment

The rats in the SCA + HBO group were placed into experimental HBO chambers (Holywell Neopren, Belgrade, Serbia) and exposed to 100% oxygen according to the following protocol: 10 min compression, 2.5 atmospheres absolute (ATA), for 60 min, and 10 min decompression. In addition, hyperbaric oxygen treatment (HBOT) was performed once daily for 10 successive days. This protocol represents the hyperbaric oxygen treatment that is routinely used in the clinical setting of The Centre for Hyperbaric Medicine, Belgrade, Serbia [21,65].

### 4.4. Brain Tissue Preparation

After the ending of HBOT, animals from all the groups were overdosed with CO_2_ and decapitated. The brains were dissected and fixed at +4 °C in 4% paraformaldehyde overnight. After fixation, the brains were cryoprotected with immersion in graded sucrose solutions (10%, 20%, and 30% in 0.2 M phosphate buffer pH 7.4) at +4 °C followed by freezing in isopentane cooled to −80 °C. Using a cryostat, the brains were cut into 25-μm thick coronal slices. Afterward, sections at 3.12–3.84 mm anteroposterior to the bregma were mounted on glass slides, air-dried at room temperature, and maintained at −20 °C, until following procedures.

### 4.5. Immunohistochemistry and Immunofluorescence Staining

Single peroxidase immunohistochemistry was performed to visualize DCX as a marker of newborn neurons to determine the effect of SCA on the immature neurons’ vulnerability in the hippocampal DG. Heated citrate buffer (pH 6) was used as antigen retrieval. Sections were washed in PBS and then incubated in 0.3% H2O2 in methanol for 20 min to block endogenous peroxidase. Normal donkey serum (NDS; 5% solution in PBS; Sigma, Munich, Germany) was used to block unspecific binding. Sections were incubated overnight at 4 °C with an anti-DCX antibody. After using the appropriate primary and peroxidase-linked secondary antibody, the products of immunoreactions were visualized with 3′3-diaminobenzidine (DAB, Dako, Glostrup, Denmark) according to manufacturer instructions. All sections were dehydrated in graded ethanol, cleared in xylene, and mounted in DPX Mounting medium (Sigma-Aldrich, Munich, Germany).

Visualization of TUJ1, a cell marker of neurons from the early stage of neural differentiation, and Ki67, widely accepted as a cell proliferation marker, was performed using double immunofluorescent staining. First, microscopic slides were incubated in 5% NDS with 0.5% Triton X-100 (Sigma-Aldrich, Darmstadt, Germany). After that, sections were incubated overnight with the appropriate primary antibody at +4 °C and with the appropriate secondary for 2 h at room temperature. Immune complexes were visualized after incubation with the secondary antibody.

The double immunofluorescence staining with Fluoro-Jade B (FJB) and Neuronal nuclear antigen (NeuN) was performed as previously described by Parabucki et al. [11]. NeuN was used as a marker of mature neurons. Briefly, sections were first incubated overnight at +4 °C with the primary antibody, and the immune reaction was visualized with a proper secondary antibody. Then, sections were pretreated with a 0.06% potassium permanganate solution for 5 min and incubated with 0.0004% solution of FJB (Chemicon International, Temecula, CA, USA) dissolved in 0.1% acetic acid for 20 min. In addition, sections were counterstained with DAPI (Invitrogen, Grand Island, NY, USA).

The list of the used primary and secondary antibodies is shown in Table 2.

### 4.6. Quantification of Immunoreactive Cells

Quantification of DCX+ cells was done along the length of the SGZ in the inner and separately in the outer blade of the right dentate gyrus [65]. At the micrographs, DCX-positive cells were easily noticeable and were counted manually. The ImageJ open-source platform (National Institutes of Health, USA; http://imagej.nih.gov/ij/download.html, accessed on 30 January 2022) was used to determine the length of the SGZ. The number of marked cells was expressed per 1 mm of the length of the SGZ.

DCX+, Ki67+, and DCX+/Ki67+ cells were quantified along the entire length of the SGZ of the DG in control, SCA, and SCA + HBO brain sections of the DG. Two independent observers manually counted the total number of single and double-positive cells at corresponding channels using Adobe Photoshop Creative Cloud (Version 14.0). The percentage of single- or double-positive cell populations was also calculated and presented.

NeuN immunoreactivity was quantified in an area of interest, which was defined within the inner and outer blades of the right DG (180 × 180 pixels). Raw immunofluorescent micrographs of the DG were taken under the same conditions at a 20× magnification using a Carl Zeiss AxioVert microscope (Zeiss, Gottingen, Germany) and then used to measure integrated fluorescence density (Figure S1). Integrated density was calculated separately for the inner and outer blades of the DG. After conversion into an 8-bit grayscale format, post-image processing was conducted using ImageJ open-source platform. For more details, see the Supplementary Material.

All images of the selected neurons, placed in the outer blade, were taken under 40× magnification for graphic processing. A total of five neurons were studied for each animal in different experimental groups. These images have been processed in the ImageJ open-source platform to analyze the dendritic arborization and total length of the dendrites in the obtained binary images. We counted the number of branching points, dendrite terminals, and segments to quantify dendritic arborization in each neuron. After converting the taken pictures into binary and skeletonized images for measuring the total dendritic length, we used ImageJ macro called measure skeleton length. The average segmental length represents a ratio between the total dendritic length and the number of segments [66].

### 4.7. Statistical Analysis

Statistical Package for the Social Sciences (SPSS; IBM, version 22.0, Armonk, NY, USA) was used for the data analysis. First, the normal distribution of data was tested using the Shapiro-Wilk test. All values are expressed as mean ± standard deviation (SD). Differences between the groups were estimated using One-way ANOVA with Tukey’s multiple comparisons post hoc test. Group differences were assessed using the Independent- Samples T-test. Statistical significance was set at *p* < 0.05, *p* < 0.01, and *p* < 0.001.

## Figures and Tables

**Table 1:** The number of branching points, dendrite total length, and average segment length in the neurons of control, SCA and HBOT brain sections.

	C	SCA	SCA + HBO	*p*		
				SCA vs. C	SCA + HBO vs. C	SCA + HBO vs. SCA
DTL	261.08 ± 24.39	148.60 ± 21.25	211.51 ± 28.40	<0.001	<0.01	<0.01
ASL	19.08 ± 1.70	25.41 ± 2.84	20.46 ± 3.67	<0.01	0.69	<0.05
BP	6.56 ± 0.96	2.57 ± 0.53	4.83 ± 0.35	<0.001	<0.01	<0.001

All values are shown as mean ± SD. The level of significance was determined using One-way ANOVA with Tukey’s multiple comparisons post hoc test. DTL—dendrite total length in µm, ASL—average segment length in µm, BP—branching points. C—Control, SCA—Suction cortical ablation, and SCA + HBO—SCA animals treated with hyperbaric oxygen.

**Table 2:** The list of the primary and secondary antibodies used for immunohistochemistry and immunofluorescence staining.

Antibody	Source	Dilution	Company
doublecortin	Goat	1:200	Santa Cruz Biotechnology, Santa Cruz, CA, USA
TUJ1	mouse	1:400	Abcam, Cambridge, MA, USA
NeuN	mouse	1:200	Milipore, Burlington, MA, USA
Ki67	rabbit	1:100	Vector Laboratories, Burlingame, CA, USA
anti-goat HRP conjugated IgG	donkey	1:200	Santa Cruz Biotechnology, Santa Cruz, CA, USA
anti-goat Alexa Fluor 488	donkey	1:200	Invitrogen (Eugene, OR, USA)
anti-mouse Alexa Fluor 555	donkey	1:200	Invitrogen (Eugene, OR, USA)
anti-rabbit Alexa Fluor 555	donkey	1:200	Invitrogen (Eugene, OR, USA)

All micrographs of stained sections were made using a Carl Zeiss AxioVert microscope (Zeiss, Gottingen, Germany) at the following magnifications: 5×, 10×, 20×, 40×, and 63×.

## Data Availability

The data presented in this study are available in article and Supplementary Material.

## Supplementary Materials

The following supporting information can be downloaded at: https://www.mdpi.com/article/10.3390/ijms24054261/s1.
