# Supplementary material for: Hyperbaric Oxygenation Prevents Loss of Immature Neurons in the Adult Hippocampal Dentate Gyrus Following Brain Injury
Source: Int J Mol Sci. 2023 Feb 21;24(5):4261. doi: 10.3390/ijms24054261 (PMC10002298; doi:10.3390/ijms24054261)
Supplement: Supplementary file 1 [file ijms-24-04261-s001.zip › ijms-2163374-supplementary.pdf]

SUPPLEMENTARY MATERIALS

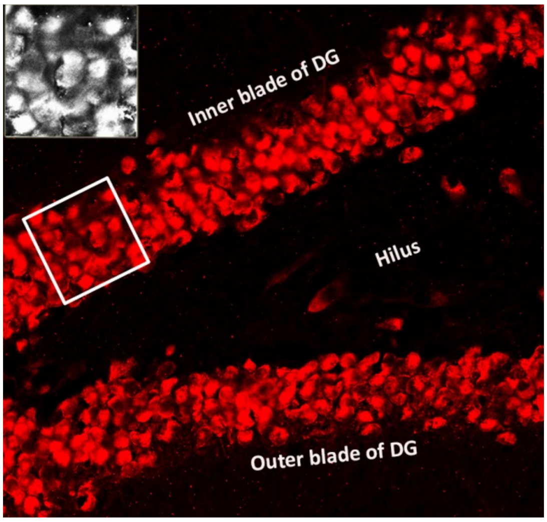

Figure S1. Quantification of NeuN fluorescence intensity

Standard digital images of the dentate gyrus were obtained under the same conditions at a 20x magnification using a Carl Zeiss AxioVert microscope (Zeiss, Gottingen, Germany). An area (180 x 180 pixels) of interest was defined within the inner and outer blade of the dentate gyrus (white square). Integrated density was calculated separately for the inner and outer blade of the dentate gyrus. After conversion into 8-bit grayscale format (enlarged micrograph in the left upper corner), post-image processing was performed using ImageJ software (National Institutes of Health, USA; http://imagej.nih.gov/ij/download.html). Magnification 20x.

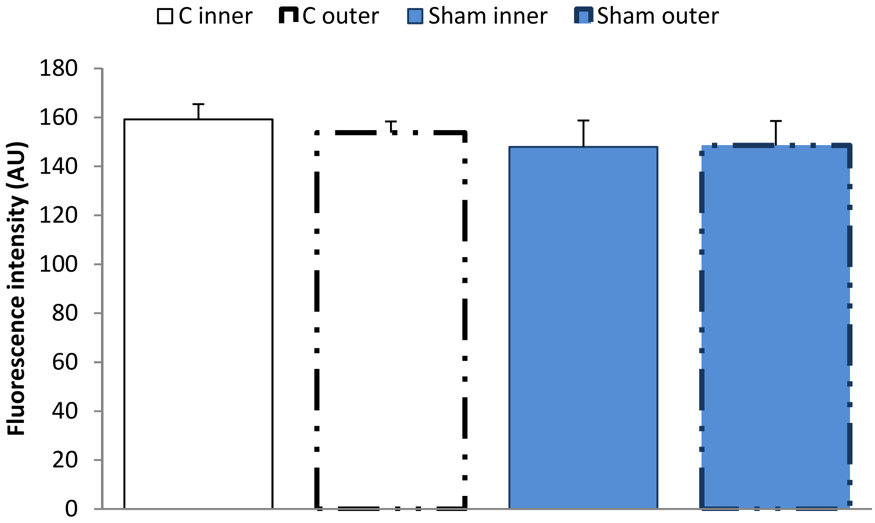

Figure S2. NeuN fluorescence signal intensity (in arbitrary units, AU) in the inner and outer blade of the hippocampal dentate gyrus in the control (C, white bars) and Sham (blue bars) group. NeuN fluorescence signal intensity was quantified in the inner and outer blade of DG separately according to the procedure given in Figure S1.

Bars represent mean ± SD. Level of significance between groups was analyzed using One-way ANOVA with Tukey's multiple comparisons post hoc test. The level of significance within the group between the inner and outer layer was analyzed using the Independent-Samples T test. No statistically significant difference in the NeuN fluorescence signal intensity within the inner vs. outer blade and between the control and sham group was observed. **Control group**: C inner vs. C outer, p = 0.114; **Sham group**: Sham inner vs. Sham outer, p = 0.921; **Control vs. Sham group**: C inner vs. Sham inner, p = 0.206; C outer vs. Sham outer, p = 0.718)

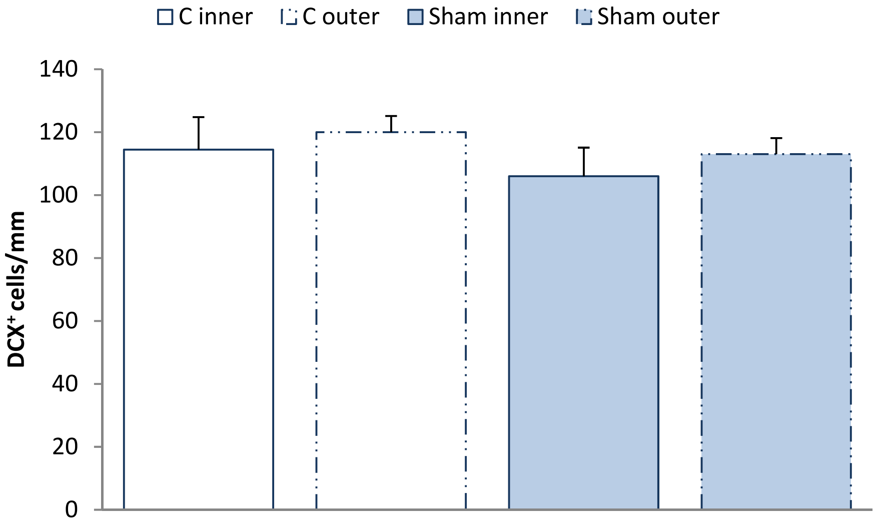

Figure S3. The number of doublecortin (DCX)-positive cells within the subgranular zone (SGZ) in the inner and outer blade of the hippocampal dentate gyrus (DG) in the control (C, white bars) and Sham (blue bars) group. DCX-positive cells were quantified along the SGZ in the inner and outer blade of DG separately. The micrographs of DG were taken using a Carl Zeiss AxioVert microscope (Zeiss, Gottingen, Germany). In those micrographs, DCX-positive cells were easily noticeable and were counted manually by two independent observers. Bars represent mean ± SD. The level of significance was analyzed using One-way ANOVA with Tukey's multiple comparisons post hoc test The level of significance within the group between the inner and outer layer was analyzed using the Independent-Samples T test. No statistically significant difference in the number of DCX-positive cells in SGZ of the inner vs. outer blade and between the control and sham group was observed. **Control group**: C inner vs. C outer, p = 0.191; **Sham group**: Sham inner vs. Sham outer, p = 0.133; **Control vs. Sham group**: C inner vs. Sham inner, p = 0.582; C outer vs. Sham outer, p = 0.520)
